# A seven-lncRNA signature predicts overall survival in esophageal squamous cell carcinoma

**DOI:** 10.1038/s41598-018-27307-2

**Published:** 2018-06-11

**Authors:** Yu Mao, Zhanzhao Fu, Yunjie Zhang, Lixin Dong, Yanqiu Zhang, Qiang Zhang, Xin Li, Jia Liu

**Affiliations:** 1 Department of Oncology, The First Hospital of Qinhuangdao, Qinhuangdao, Hebei China; 2 Institute of basic medical sciences, Qilu Hospital, Shandong University, Jinan, Shandong China

## Abstract

Esophageal squamous cell carcinoma (ESCC) is one of the most common types of cancer and the leading causes of cancer-related mortality worldwide, especially in Eastern Asia. Here, we downloaded the microarray data of lncRNA expression profiles of ESCC patients from Gene Expression Omnibus (GEO) and The Cancer Genome Atlas (TCGA) data sets and divided into training, validation and test set. The random survival forest (RSF) algorithm and Cox regression analysis were applied to identify a seven-lncRNA signature. Then the predictive ability of the seven-lncRNA signature was evaluated in the validation and test set using Kaplan-Meier test, time-dependent receiver operating characteristic (ROC) curves and dynamic area under curve (AUC). Stratified analysis and multivariate Cox regression also demonstrated the independence of the signature in prognosis prediction from other clinical factors. Besides, the predict accuracy of lncRNA signature was much better than that of tumor-node-metastasis (TNM) stage in all the three sets. LncRNA combined with TNM displayed better prognostic predict ability than either alone. The role of LINC00173 from the signature in modulating the proliferation and cell cycle of ESCC cells was also observed. These results indicated that this seven-lncRNA signature could be used as an independent prognostic biomarker for prognosis prediction of patients with ESCC.

## Introduction

Esophageal cancer ranks the 8th most common type of cancer worldwide and the 6th leading cause of cancer mortality^1^. There are two main histological types of esophageal cancer: esophageal adenocarcinoma (EAC) and esophageal squamous cell carcinoma (ESCC). These two cancer types differ from each other in terms of causes, incidence patterns and biology features. Although the incidence of EAC is increasing rapidly in Western countries, ESCC still remains dominant in East Asian^2^. Besides, the overall 5-year survival rate of ESCC remains extremely poor with a high probability of recurrence and metastasis^3^. Despite the tumor-node-metastasis (TNM) system has been widely used as prognostic factors, substantial differences exist in survival among patients within the same clinical stage, as a result of the heterogeneous of ESCC. Hence, there is an urgent need for fully comprehensive research into the crucial molecular mechanisms associated with the prognosis of ESCC.

Long non-coding RNAs (lncRNAs) are defined as RNA transcripts longer than 200 nucleotides that lack protein-coding abilities^4^. Nowadays, lncRNAs have attracted increasing scientific interest and recent evidence revealed their role as an important molecular players in modulating diverse biological processes. They have been reported to regulate gene expression through chromatin modification, transcriptional and post-transcriptional processing^5^. For instance, the well-known lncRNA HOTAIR induce the transcriptional repression of HOX loci and genome-wide retargeting of PRC2 (polycomb repressive complex 2) which results in altered histone H3K27 methylation and metastasis-related gene expression^4^.

In addition to the regulation of biological process, recent studies have revealed that lncRNAs can serve as potential prognostic biomarkers and several prognostic lncRNA signatures have been identified and validated in many cancer types, such as gastric cancer, colorectal cancer and clear cell renal cell carcinoma^6–8^. However, the prognostic role of lncRNA in ESCC remain largely unknown, mainly due to the lack of the comprehensive and systemic analysis of lncRNA profiling analysis in ESCC^9^. Presently, since the recent release of gene expression data and related prognosis information in Gene Expression Omnibus (GEO) and The Cancer Genome Atlas (TCGA), we mined the LncRNA data from the GEO and conducted lncRNA profiling on ESCC patients. We identified a prognostic, seven-lncRNA signature for ESCC from the training set of GEO and validated its prognostic value in two independent test sets including the GEO validation set and another independent TCGA test set.

## Results

### Derivation of prognostic lncRNAs from the training set

By subjecting the lncRNA expression data from GEO training set to RSF algorithm and univariable Cox regression analysis, a set of seven lncRNAs that significantly correlated with patients’ overall survival was firstly identified. The list of seven prognostic lncRNAs and their obtained specific values including permutation P values, hazard ratios and coefficients were shown in Table 1. Among these genes, four lncRNAs (RP5-1172N10.2, RP11-579D7.4, RP11-89N17.4, LA16c-325D7.2) had positive coefficients which suggested that higher expression level was associated with shorter survival and three (RP1-251M9.2, RP11-259O2.2, LINC00173) had negative coefficients suggested that higher levels of expression were related with longer survival.

**Table 1:** LncRNAs significantly associated with the overall survival in the training set.

Gene symbol	Permutation P value	Hazard ratio	Coeffcient
RP5-1172N10.2	5.30E-05	5.3005	1.6678
RP11-89N17.4	3.6 E-05	3.3800	1.2179
LA16c-325D7.2	2.6 E-04	1.6159	0.4799
RP11-579D7.4	2.3 E-04	1.1699	0.1570
RP1-251M9.2	9.10E-05	0.1200	−2.1202
RP11-259O2.2	4.6 E-05	0.8100	−0.2107
LINC00173	1.3 E-04	0.8500	−0.1625

Risk score = (1.6678 × expression level of RP5-1172N10.2) + (1.2179 × expression level of RP11-89N17.4) + (0.4799 × expression level of LA16c-325D7.2) + (0.1570 × expression level of RP11-579D7.4) + (−2.1202 × expression level of RP1-251M9.2) + (−0.2107 × expression level of RP11-259O2.2) + (−0.1625 × expression level of LINC00173).

### The seven-lncRNA signature predicts the survival of patients with ESCC

**Figure 1:**
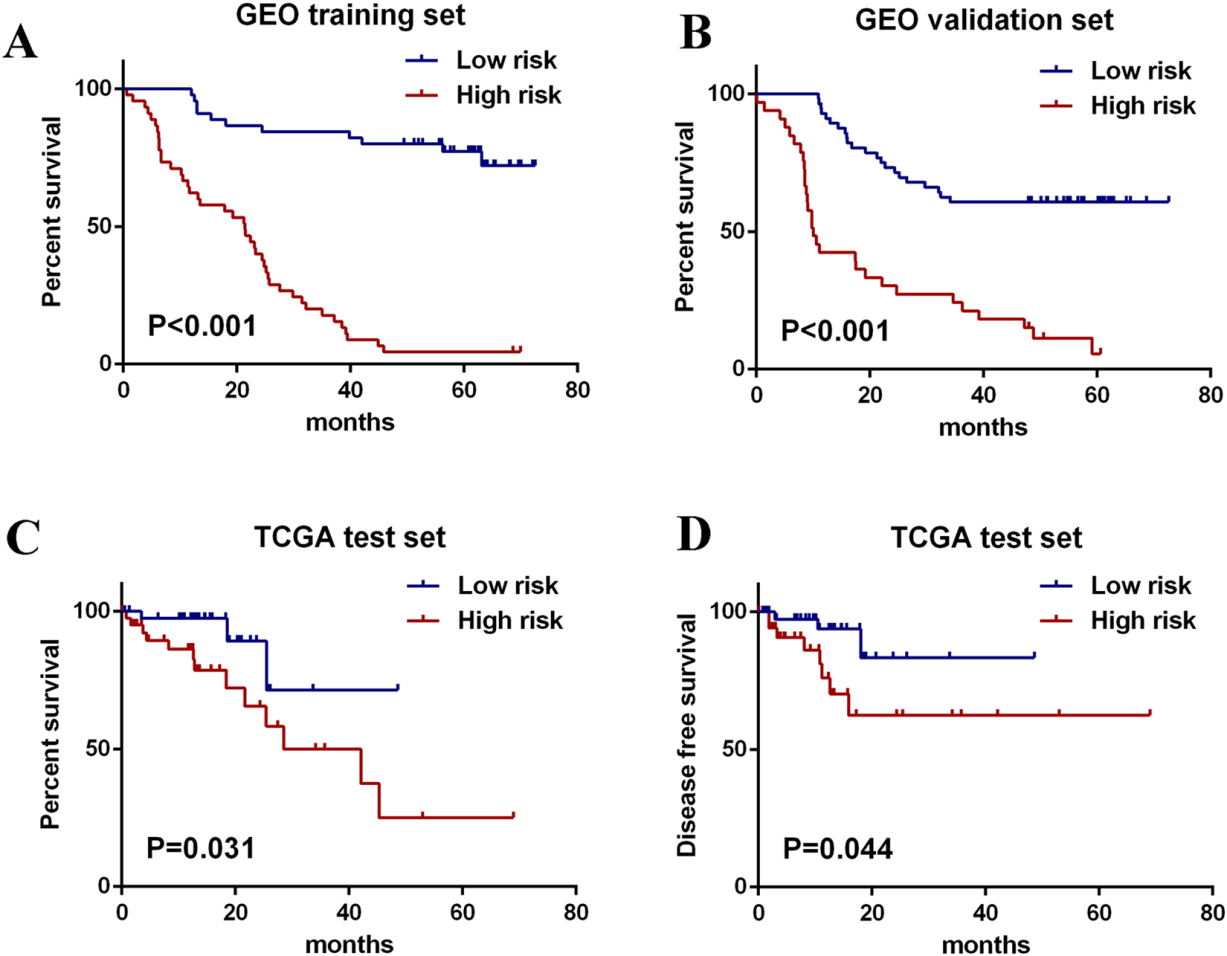
Kaplan-Meier estimates of the OS and DFS in GEO and TCGA patients using the seven-lncRNA signature. The Kaplan-Meier curves were used to visualize and compare the OS of the low-risk versus high-risk group in GEO training set (**A**), GEO validation set (**B**) and TCGA test set (**C**). The Kaplan-Meier curves for the DFS of low-risk versus high-risk group in TCGA test set was also plotted (**D**).

A risk score formula based on the expression level and coefficient of seven lncRNAs was created as follows: Risk score = (1.6678 × expression level of RP5-1172N10.2) + (1.2179 × expression level of RP11-89N17.4) + (0.4799 × expression level of LA16c-325D7.2) + (0.1570 × expression level of RP11-579D7.4) + (−2.1202 × expression level of RP1-251M9.2) + (−0.2107 × expression level of RP11-259O2.2) + (−0.1625 × expression level of LINC00173). Next, the lncRNA signature based risk score for each patient in the training set was calculated, and patients in the cohort was assigned into high-risk group (n = 45) and low risk group (n = 45) according to the median risk score value as the cutoff point. Kaplan-Meier curves showed that patients in the high-risk group had significantly shorter OS than those in the low-risk group (log-rank test p < 0.001) (Fig. 1A).

The predictive efficiency of the seven-lncRNA signature in GEO validation set with 89 patients was then evaluated. By using the same model and criteria, patients in the validation set was classified into high-risk (n = 56) and low-risk groups (n = 33). Similar with that in training set, the overall survival of the high-risk group patients was significantly worse than that of low-risk group patients (p < 0.001) (Fig. 1B). Risk score-based classification of the external test set from TCGA also yielded similar results as shown in Fig. 1C. Besides, the value of seven-lncRNA signature in predicting the disease free survival (DFS) was also detectable according to the Kaplan-Meier curves of TCGA cohort as shown in Fig. 1D.

**Figure 2:**
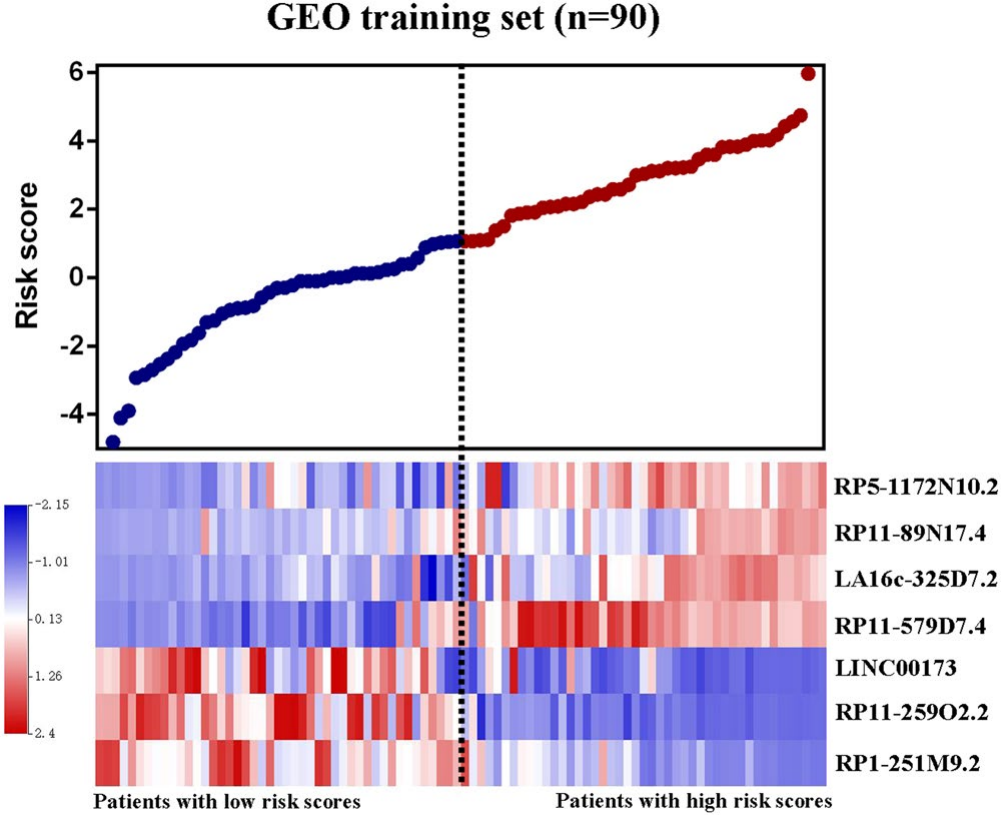
Risk score analysis of GEO training set. The distribution of seven-lncRNA risk score and heat maps of the corresponding lncRNA expression level.

The distribution of the risk score, overall survival status along with the corresponding expression profiles of seven lncRNAs from the GEO training cohort were showed in Fig. 2, which were ranked according to the risk score value. Patients with higher-risk scores tended to have higher expression level of risky lncRNAs (RP5-1172N10.2, RP11-89N17.4, LA16c-325D7.2, RP11-579D7.4). On the contrary, patients with lower-risk scores tended to have higher expression level of protective lncRNAs (RP1-251M9.2, RP11-259O2.2, LINC00173) (Fig. 2).

### Prognostic value of the seven-lncRNA signature is independent of clinical and pathological factors

To explore the independence of seven-lncRNA signature from other clinical or pathological factors in prognosis prediction, multivariable Cox regression analysis was performed. Variables included age, sex, tobacco use, pathology grade, TNM stage and lncRNA signature were included into the multivariable Cox regression model. According to the results of multivariable Cox regression in training set, lncRNA signature were significantly associated with overall survival of the patients as a continuous variable, which was in consistence with that in the TCGA test set. In GEO validation set, the seven-lncRNA signature and TNM stage were both significant prognostic factors for patients with ESCC (Table 2). Hence, the results of the multivariable Cox regression analysis suggested the independence of lncRNA signature in the overall survival prediction from other clinical and pathological factors for patients with ESCC.

**Table 2:** Univariable and multivariable Cox regression analysis in each data set.

Variables	Univariable model			Multivariable model		
	HR	95% CI of HR	P value	HR	95% CI of HR	P value
**GEO training set (N** = **90)**						
Gender	2.089	0.147 to 3.805	0.162	0.753	0.280 to 2.028	0.575
Age	1.824	1.058 to 3.145	0.031	1.305	0.721 to 2.362	0.379
Tobacco use	1.884	1.088 to 3.263	0.024	1.439	0.572 to 3.621	0.439
Pathology grade	1.059	0.712 to 1.577	0.777	1.093	0.690 to 1.732	0.705
TNM stage	2.814	1.721 to 4.602	0.003	1.365	0.815 to 2.287	0.237
LncRNA signature	2.012	1.668 to 2.428	<0.001	1.892	1.550 to 2.309	0.001
**GEO validation set (N** = **89)**						
Gender	0.703	0.317 to 1.560	0.386	0.399	0.154 to 1.032	0.058
Age	1.628	0.941 to 2.816	0.081	2.426	0.302 to 4.519	0.205
Tobacco use	1.268	0.735 to 2.190	0.394	1.499	0.799 to 2.812	0.208
Pathology grade	0.536	0.357 to 0.805	0.003	0.663	0.429 to 1.026	0.065
TNM stage	2.429	1.420 to 4.155	0.001	2.205	1.257 to 3.868	0.006
LncRNA signature	2.112	1.692 to 2.636	<0.001	2.052	1.623 to 2.595	0.001
**TCGA test set (N** = **81)**						
Gender	0.027	0.000 to 3.411	0.144	0.001	0.000 to 4.401	0.977
Age	1.313	0.471 to 3.660	0.603	1.737	0.454 to 6.638	0.420
Tobacco use	0.312	0.071 to 1.380	0.125	0.675	0.075 to 6.101	0.727
Pathology grade	1.158	0.527 to 2.545	0.716	0.510	0.168 to 1.546	0.234
TNM stage	1.820	0.963 to 3.438	0.065	0.730	0.276 to 1.928	0.525
LncRNA signature	2.193	1.369 to 3.514	0.001	2.613	1.230 to 5.550	0.012

In Cox regression analysis, Tumor grade, TNM and LncRNA signature were evaluated as continuous variables. Gender and Tobacco use were evaluated as category variable.

### The lncRNA signature has prognostic predictive value within TNM stages

**Figure 3:**
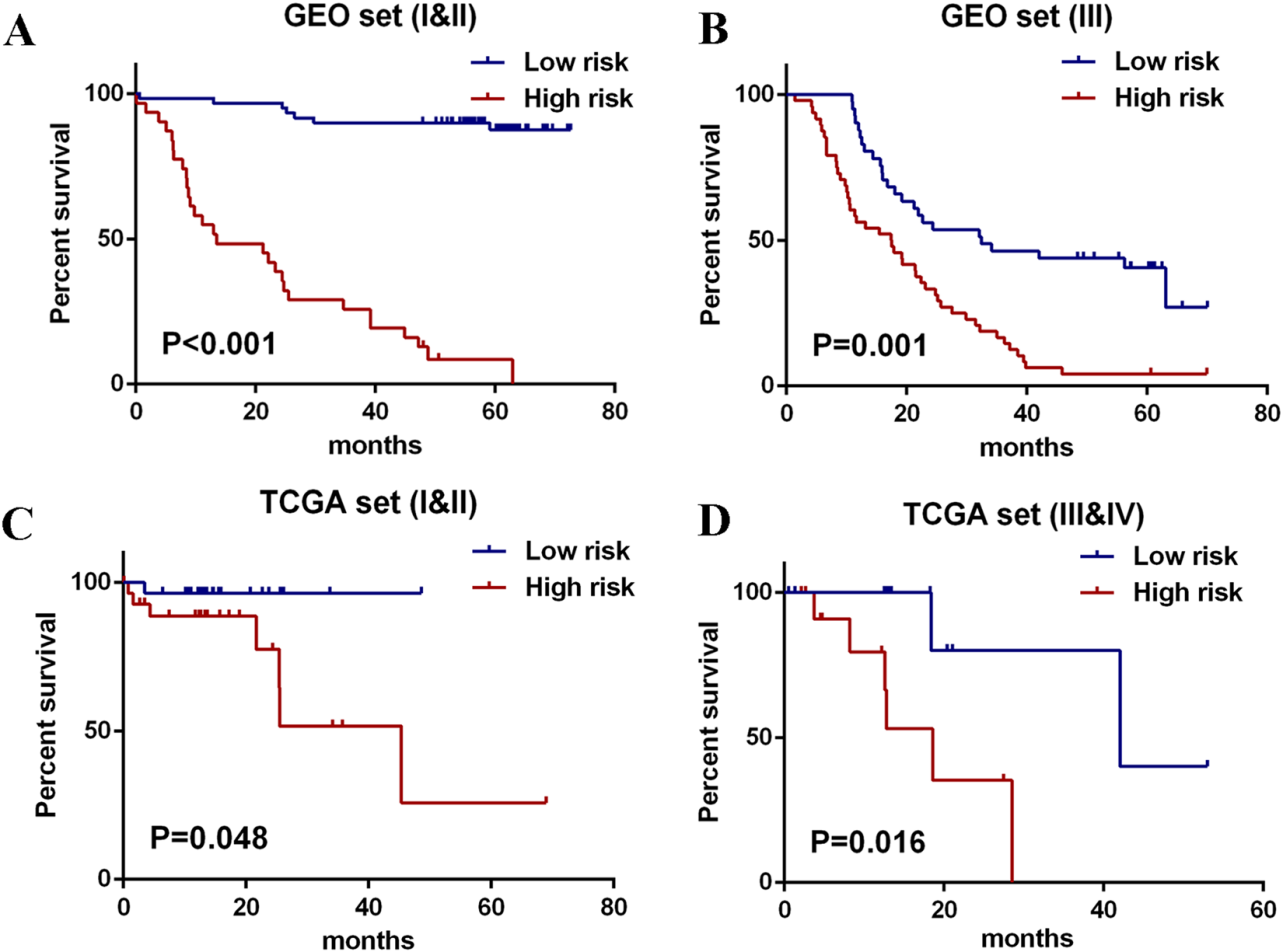
Kaplan-Meier estimates of the OS in GEO and TCGA patients using the seven-lncRNA signature, stratified by TNM stage. (**A**) Patients with ESCC of TNM stage I&II in GEO entire set. (**B**) Patients with ESCC of TNM stage III in GEO entire set. (**C**) Patients with ESCC of TNM stage I&II in TCGA test set. (**D**) Patients with ESCC of TNM stage III&IV in TCGA test set.

Because of limited sample size in each TNM stage, patients in the entire GEO set were divided into low TNM stage (I & II) and high TNM stage(III). In external TCGA test set, patients were divided into low TNM stage (I & II) and high TNM stage (III & IV). Then a stratified analysis in low and high TNM stage was carried out. The log-rank test suggested that the seven-lncRNA signature could identify patients with low and high TNM stage in both TCGA and GEO set. (Fig. 3)

### Evaluation and comparison of the prognostic accuracy between the lncRNA signature and TNM

In evaluating sensitivity and specificity of a model, it comes to address the basic question: How well does the model discriminate who are likely to die from who are likely to survive at the given time point? Furthermore, we consider whether the accuracy of the model changes over time.

**Figure 4:**
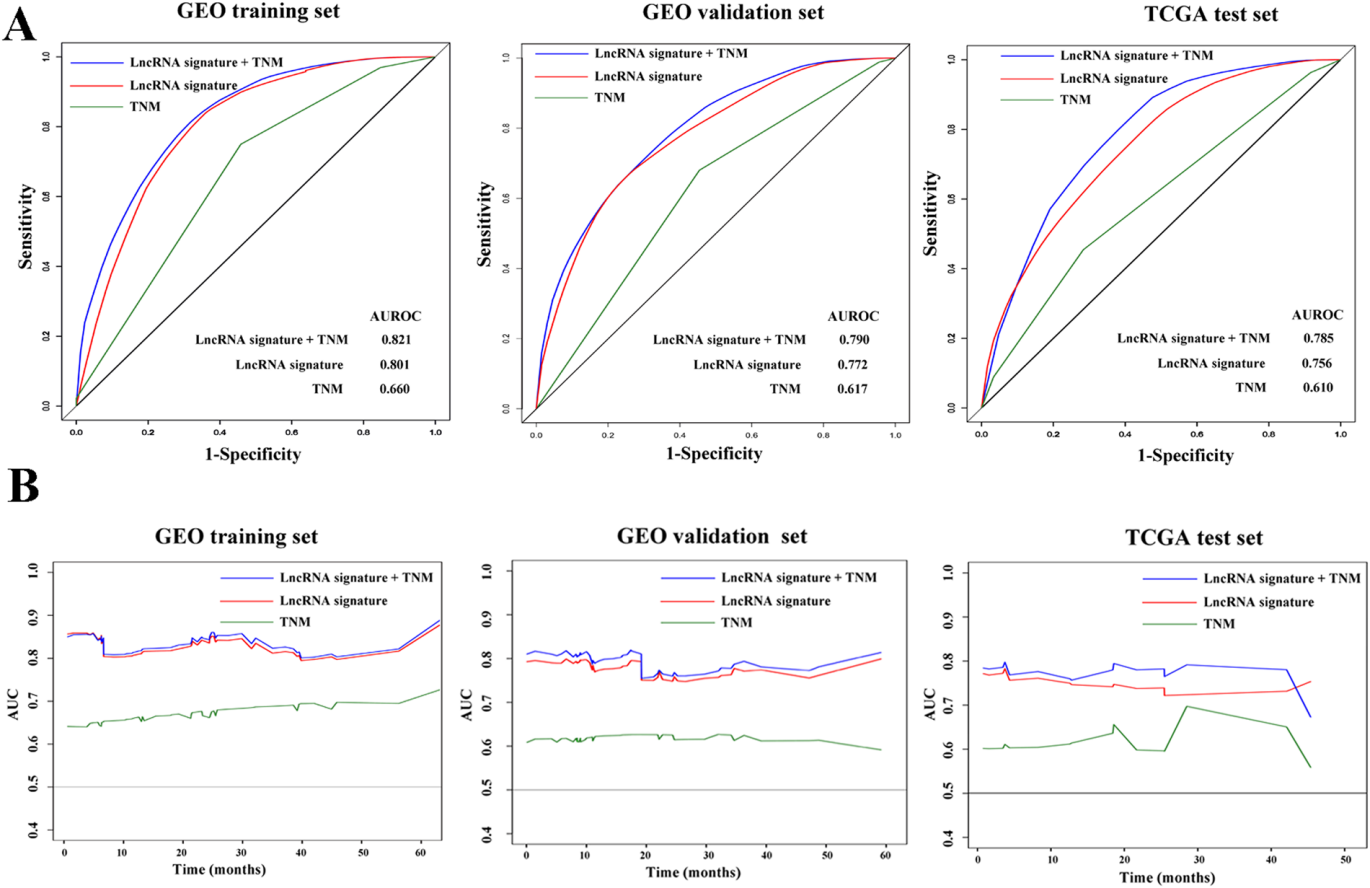
Prognostic value evaluation of TNM stage the lncRNA signature. The time-dependent ROC curves on the 12th month of follow up were plotted to assess the prognostic efficiency of TNM stage, lncRNA signature and a variable combining both. (**A**) The dynamic AUC line for TNM stage, lncRNA signature and the combined variable were delineated.

Firstly, we constructed time-dependent time-dependent receiver operating characteristic (ROC) curves and assessed the dynamic area under curve (AUC) to evaluate the sensitivity and specificity of variables in classifying death and survival on the 12th month of follow up. TNM stage, lncRNA signature and a variable combining both were included into the comparison. In GEO training set, predictive ability of the combined variable was better than LncRNA signature and TNM stage alone on the 12th month. lncRNA signature showed a more efficient predictive ability than TNM stage. Similar results were also found in both in GEO validation set and TCGA test set (Fig. 4A).

In order to depict the dynamic accuracy of the model over time, the dynamic AUC of each time-dependent ROC curves at continuous time point were calculated and plotted as line chart in Fig. 4B. In GEO training set, the combined variable has good discriminatory capacity for distinguishing those patients who die at every time point from those who live beyond the time point, with dynamic AUC estimates exceeding 0.80. The accuracy of combined variable was better than lncRNA signature or TNM stage alone. In GEO validation set, the combined variable displayed better prognostic predict ability of overall survival than lncRNA signature or TNM stage alone with average dynamic AUC estimates exceeding 0.75. In external TCGA test set, the dynamic AUC line for the combined variable is approximately 0.10 units upon that of lncRNA signature alone. Due to the limited sample size of the TCGA test set with follow up times more than 40 months, the three dynamic AUC lines fluctuated violently and a cross was found between the dynamic AUC line of combined variable and LncRNA signature. Besides, the predict accuracy of LncRNA signature was much better than that of TNM stage in all the three sets.

### Functional enrichment analysis of genes correlated with the lncRNAs in signature

**Figure 5:**
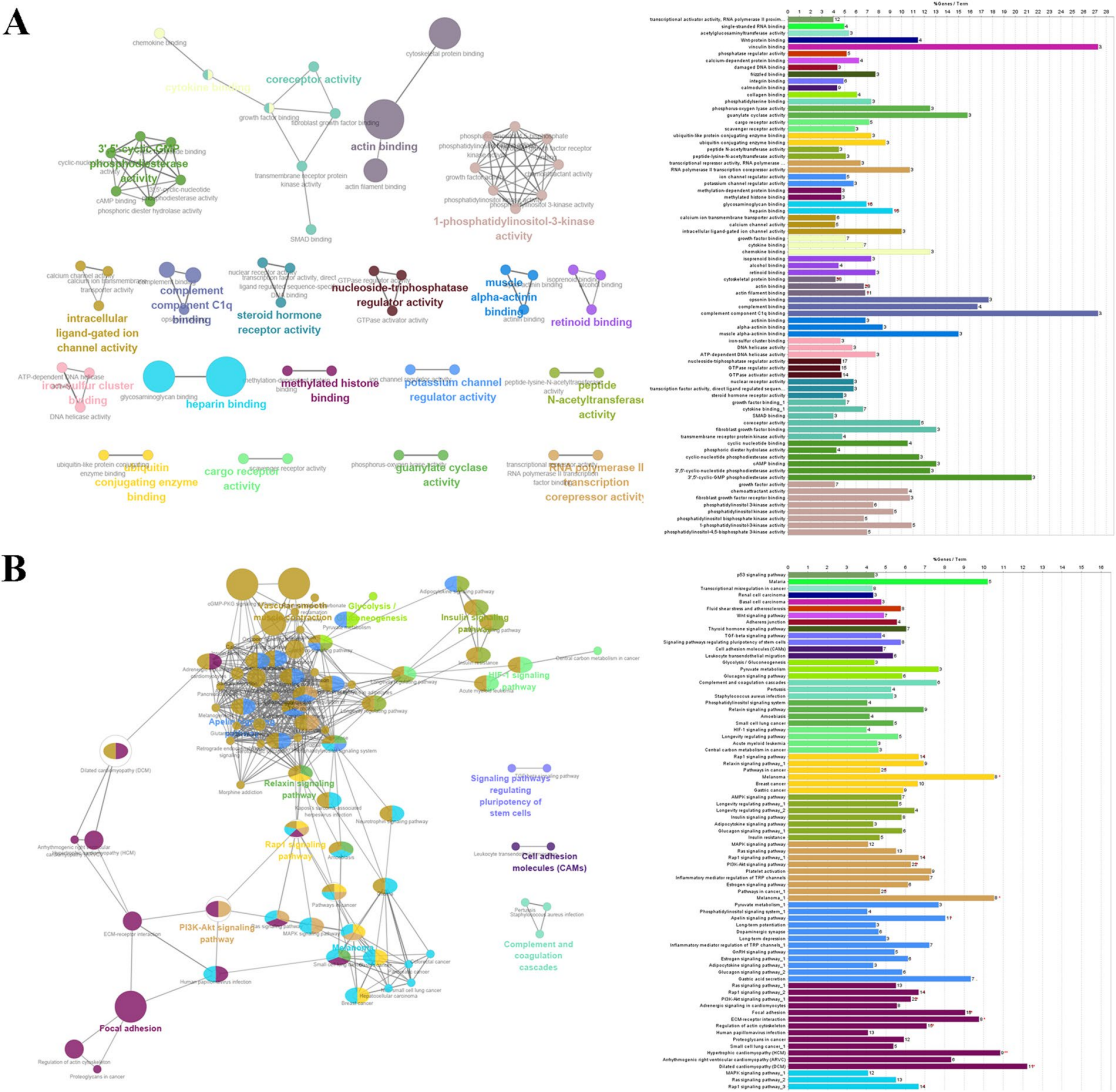
Functional enrichment analysis depicted the biological pathways and processes associated with correlated genes. The results of GO biological process enrichment (**A**) and KEGG signaling pathways analysis (**B**).

We next sought to identify the biological pathways and processes correlated with the seven-lncRNA signature. According to the theory of competing endogenous RNAs (ceRNAs), lncRNAs act as rheostats that fine-tune gene expression and maintain the functional balance of various gene networks^10^. Hence, we analyzed the correlation between their expression values and the mRNAs in the TCGA test set. Genes correlated with the seven lncRNAs with pearson correlation coefficient >0.60 or <−0.40 were summarized into the cohort. Then the cohort were put into gene ontology (GO) biological process enrichment and KEGG (Kyoto Encyclopedia of Genes and Genomes) signaling pathways analysis. According to the results, these genes play important roles in cancer related biological processes such as cell cycle regulation and histone methylation and signaling pathways such as PI3K-Akt and HIF-1 pathway. These analysis suggested that the lncRNAs of the signature may regulate the tumorigenesis and progression of ESCC via acting as the ceRNA and modulate the expression of their targeting genes (Fig. 5).

### Knock down of LINC00173 facilitates the cell proliferation and cell cycle of ESCC cells

**Figure 6:**
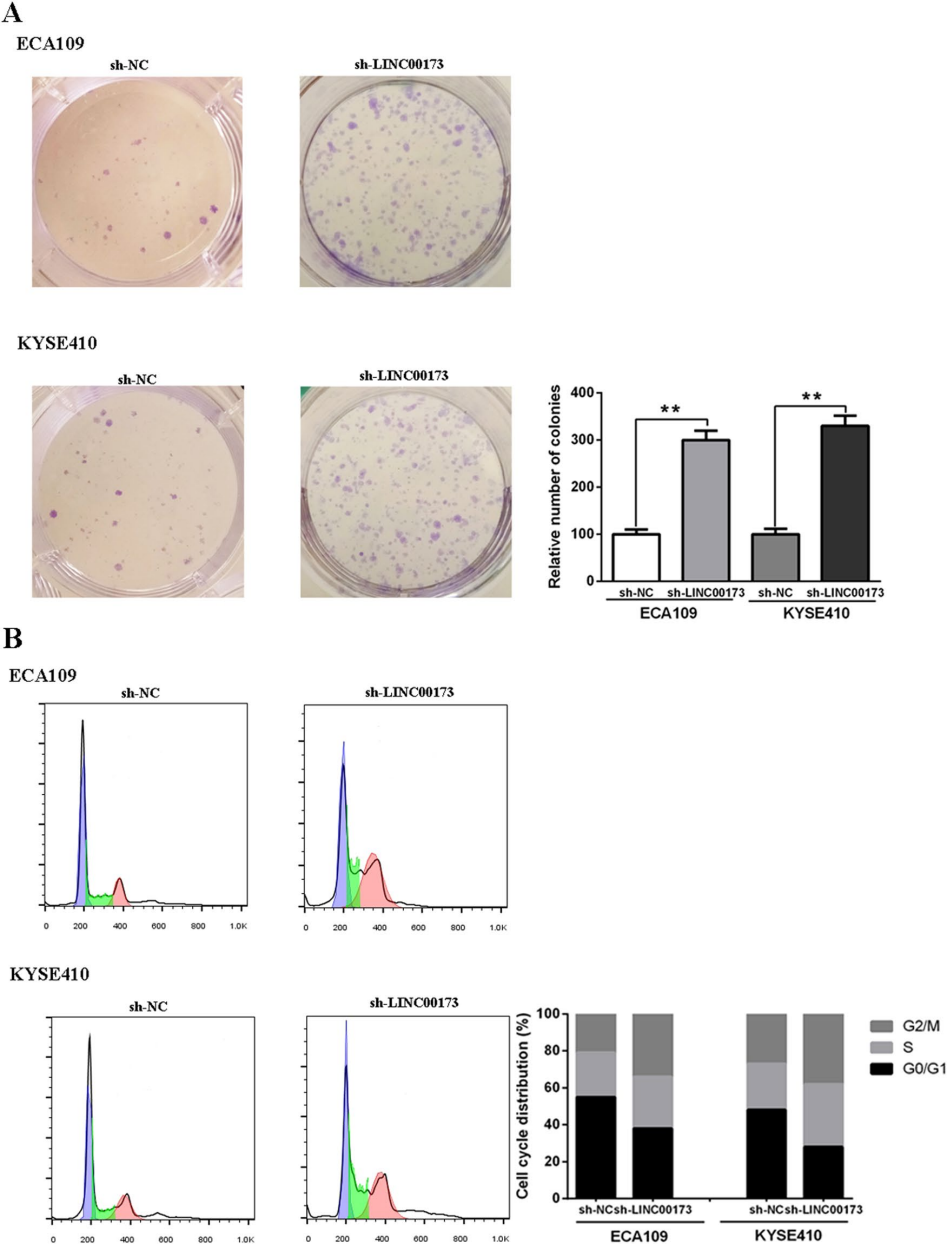
Regulatory role of LINC00173 in ESCC cell lines. Colony formation assays showed that the knockdown of LINC00173 boosted the colony number (**A**). Cell cycle analysis demonstrated that LINC00173 knockdown led to a decreased G1/G0 population (**B**).

Among the LncRNAs aforementioned, the role of LINC00173 in modulating the proliferation and differentiation of granulocytes has been previously validated^11^. Here, we further explored its role in ESCC cell lines by transfecting sh-LINC00173 to knock down LINC00173 expression. Colony formation assays showed that the knockdown of LINC00173 boosted the colony number (Fig. 6A). Cell cycle analysis demonstrated that LINC00173 knockdown led to a decreased G1/G0 population (Fig. 6B).

## Discussion

For most type of cancers, including ESCC, TNM stage still act as the main reference to direct the treatment strategies and is used as a prognostic predictor. However, as a result of the heterogeneity of cancer at the molecular and genetic levels, the clinical outcome and prognosis of patients diverse even if they are in the same stage and received similar treatment^12,13^. Currently, with the advancements of high-throughput technologies including microarray and RNA sequencing, gene expression profiling has become a powerful technique to identify the molecular biomarkers of esophageal cancer phenotypes or prognosis^14^. Multigene signatures which is designed to analyze the activity of a group of genes that strongly correlated with the behavior of the cancer have been marketed already, such as Oncotype DX Test for breast cancer or ColoPrint for colon cancer. These signatures can be applied to help cancer treatment and prognosis management^15^.

Growing evidence suggests that the aberrant expression of specific lncRNAs may acts as major contributor to tumorigenesis and intimately correlated with tumor progression. Recent studies have focused on the role of specific lncRNAs which serves as independent markers for predicting prognosis in disease such as colorectal cancer, glioma and pancreatic cancer^8,15–17^. Although a series previous articles have revealed the potential value of lncRNAs in ESCC prognosis predicting, such as HOTAIR^18^, CCAT2^19^ and MALAT1^20,21^. However, the use of the combination of lncRNAs in predicting ESCC prognosis have not been elucidated clearly.

Here, we analyzed the lncRNAs expression profiles of patients with ESCC downloaded from GEO and identified a robust seven-lncRNAs signature associated with overall survival which was independent of classical prognostic factors and molecular subtypes. The prognostic value of the lncRNAs signature was further validated in the GEO validation set and an external independent test set from TCGA. When we tried to identify the prognosis related lncRNAs from GEO training set which is the high-throughput biological data, the common problem, ‘curse-of-dimensionality’ (small sample size combined with a very large number of genes) was taken into consideration. In view of this, we applied the RSF algorithm to pick out lncRNAs and narrow down the high dimension. The random forests method bases predictions on majority voting of a collection of decision trees which exploits maximal sub-trees for effective variable selection. The criteria of gene importance is used to filter the original gene set iteratively which results in good performance in feature selection^22^. The random sampling and ensemble strategies used in the RSF method achieves greater stability and accurate predictions while running efficiently on ‘curse-of-dimensionality’ data^23^.

Next, the prognostic related lncRNAs were further selected to construct a risk score formula by Cox regression model. Cox Regression model builds a predictive model for time-to-event data. The model produces a survival function that predicts the probability that the event of interest has occurred at a given time for given values of the predictor variables. The shape of the survival function and the regression coefficients for the predictors are estimated from observed subjects; the model can then be applied to new cases that have measurements for the predictor variables^24^. After subjecting the selected genes to Cox regression analysis, a risk score formula was constructed based on their estimated regression coefficients. By applying the seven-lncRNA signature to the GEO training set, GEO validation set and TCGA test set, obvious separation was observed in the survival curves of the high-risk group and low-risk group classified by the same criteria in all three sets which indicated the high reproducibility of this lncRNA signature in ESCC. Further analysis showed that the seven-lncRNA signature was of prognostic significance no matter it was considered as a continuous variable (in multivariable Cox regression analysis) or category variable (in log-rank p test). Moreover, multivariable Cox regression and stratification analysis demonstrated that the prognostic value of the seven-lncRNA signature was independent of the TNM stage and lncRNA signature had prognostic predict ability within clinical stages.

In order to evaluate and compare the predictive efficiency, we introduced the time-dependent ROC curves and dynamic AUC which are more useful when the data is a censored survival time. A number of previous research have applied familiar binary outcome methods such as ROC curves to evaluate the specificity and sensitivity of a marker in survival prediction. Routine ROC analysis can only characterize the accuracy of a marker by focusing on the correct classification rates of the final status. However, the survival data is usually a combination of the status at the end of follow-up (binary) and the length of follow-up (continuous). The methods which estimates only the classification of binary outcome may not be extended for survival outcomes. Hence we constructed time-dependent ROC curves to assess the sensitivity and specificity of variables in classifying death and survival on the 12th month of follow up and calculate the corresponding AUC. Then the AUC for time specific ROC curves at continuous time point was calculated and further plotted as a function of time to characterize temporal changes in accuracy. In this way, we showed that the predictive accuracy of LncRNA signature were much better than that of TNM stage. Moreover, a new variable, which combined both LncRNA and TNM, displayed better prognostic predict ability of overall survival than lncRNA signature or TNM stage alone.

Despite growing studies began focus on the molecular mechanisms of lncRNA functions in malignancy, most lncRNAs are not yet functionally annotated. LncRNAs generally function as the ceRNAs which regulate gene expression through epigenetic mechanisms or posttranscriptional events such as mRNA processing and degradation^10^. Hence, we can infer the possible effect of the lncRNAs on ESCC through performing functional enrichment of their related genes^25^. The results suggested that these genes were enriched in cancer related biological process such as cell cycle regulation and histone methylation and signaling pathways such as PI3K-Akt and HIF-1 pathway. Here, we also observed the role of LINC00173 in modulating the proliferation and cell cycle of ESCC cells. Besides, the correlations between some of the LncRNAs and other tumors have already been elucidated. For instance, a recent research revealed the role of LINC00173 in the formation and maintenance of the human blood hierarchy, highlighting the function of LINC00173 in leukemia^11^. Hence, the functional enrichment analysis uncovered the underlying molecular mechanisms of these lncRNAs in playing roles in survival prediction.

The whole process of our analysis have been plotted as a flowchart in the supplementary material (Supplementary figure).

## Conclusions

In conclusion, this study reported a seven lncRNA signature to predict prognosis in ESCC patients by integrating and mining currently available microarray data. Moreover, the time-dependent ROC curves and dynamic AUC were introduced to evaluated its predictive accuracy which showed that the new variable combined both lncRNA and TNM displayed better prognostic predict ability than either alone. The functional enrichment analysis and experiments suggested that the lncRNAs in signature might be correlated with several cancer related processes and pathways, which supported the prognosis predictive ability of the lncRNAs. Future studies will focused on the validation of the signature in prospective clinical trials and the molecular mechanisms exploration and explanation of these lncRNAs.

## Methods

### The esophageal cancer patient information and data sets preparation

Microarray data of LncRNA expression from GEO data sets (GSE53622, GSE53624 and GSE53625) were downloaded and processed (http://www.ncbi.nlm.nih.gov/geo/)^22^. The LncRNA expression profiling of 179 patients with ESCC were all performed using the Agilent human lncRNA + mRNA array V.2.0 platform. Additionally, these 179 samples from GEO were randomly divided into the training set and validation set. For prognostic signature analysis, samples from GEO data set were randomly divided into training (n = 90) and validation sets (n = 89).

The genome-wide lncRNA expression profiles for ESCC patients and corresponding clinical information were downloaded from TCGA (https://tcga-data.nci.nih.gov/). After excluding patients without complete clinical and survival information, a total of 81 patients with ESCC were enrolled into this study. After alignment to the human genome (Ensembl genome browser 90), we obtained 14449 LncRNAs based on their Transcript stable ID and Gene stable ID. We determined the expression level of each lncRNA according to the value of Reads Per Kilobase of exon model per Million mapped reads (RPKM).

### LncRNA expression profiling and survival model construction

The LncRNA expression data were imported into Biometric Research Branch-Array (BRB-Array) for analysis^26^. The BRB-Array Tools utilizes the “DESeq2” R package to transform and normalize the count data^27^. The missing value were estimated using weighted K-nearest neighbors^28^. Subsequently, gene filter was conducted. Genes with the following conditions would be excluded from the set: 1. Less than 20% of expression data values have at least a 1.5-fold change in either direction from the gene’s median value. 2. More than 50% of gene expression was missing. 3. More than 50% of the intensity was less than 0.1 after normalization.

To identify the survival associated lncRNA, lncRNAs in the training set were firstly filtered through applying the random survival forest (RSF) algorithm which is a Random Survival Forest package in the variable selection function of BRB-Array Tools^23^. LncRNAs with p < 0.05 were considered as mostly associated with the prognostic classification and were applied for further analysis. Then the univariable Cox regression analysis along with a permutation test was applied to evaluate the association between the lncRNA expression and patient’s overall survival. lncRNAs with permutation p values < 0.0001, which computed based on 10,000 random permutations, were considered as significantly associated with survival and enrolled into the signature. Then a risk score formula was constructed by involving each of the selected genes, weighted by their estimated regression coefficients in the univariate Cox regression model aforementioned. The risk score of each patient was calculated according to this prognostic seven-lncRNA signature.

### Predictive accuracy evaluation of the survival model

Kaplan–Meier plotter along with log-rank p test was applied to compare the survival differences between high-risk and low-risk group. Stratified analysis and multivariate Cox regression were performed to evaluate the independence of lncRNA signature in survival prediction with other clinical variables.

Besides, time-dependent ROC (receiver operating characteristic) curves was applied to characterize the predictive accuracy of the scalar markers including lncRNA signature, TNM stage and a variable combining both. Based on time-specific versions of sensitivity and specificity calculated over risk sets, this new version of ROC curves are useful for detecting the predictive accuracy of a scalar marker when the outcome is a censored survival time. It connect the accuracy summaries to a previously proposed global concordance measure, which is a variant of Kendall’s tau. Moreover, the dynamic area under the time specific ROC curves (dynamic -AUC) can be plotted as a function of time to characterize temporal changes in accuracy^29,30^. The estimation of survival model was performed using R Package ‘risksetROC’.

### Functional enrichment

In evaluating the function of lncRNAs in signature, genes significantly related to the lncRNAs were identified via calculating the pearson correlation coefficients between seven lncRNAs and mRNAs in the data from TCGA. Genes correlated with at least one of the seven signature lncRNAs were enrolled into the analysis (Pearson correlation coefficient >0.60 or <−0.40). Functional enrichment analysis for these genes were performed and visualized using Cytoscape software with ClueGO and CluePedia Plugins^31,32^.

### Cell culture and cell proliferation assay

ESCC cell lines (ECA109 and KYSE410) were cultured in DMEM with 10% FBS (Gibco, USA) at 37 °C containing 5% CO2. For cell proliferation assay, ESCC cells were seeded into each well of the 6-well plates (500 cells/well) for 2 weeks. The colonies were stained with crystal violet for 15 min and then counted.

### Plasmid constructs and cell transfection

The shRNA that used to knock down LINC00173 (sh-LINC00173) in ESCC cells were generated by GenePharma (Shanghai, China). Transfection assays were performed using Lipofectamine 3000 Reagents (Invitrogen, USA). The transfection effciencies were assessed by RT-PCR.

### Cell cycle analysis

After the ESCC cells were fixed with ice-cold ethanol for 24 hours, they were dyed with propidium iodide/RNase buffer (BD Biosciences, USA) for 30 min in a darkplace. Then, the cells were analyzed by flow cytometry.

## Electronic supplementary material

Flowchart
