# Supplementary material for: A seven-lncRNA signature predicts overall survival in esophageal squamous cell carcinoma
Source: Sci Rep. 2018 Jun 11;8:8823. doi: 10.1038/s41598-018-27307-2 (PMC5995883; doi:10.1038/s41598-018-27307-2)
Supplement: Supplementary file 1 — Flowchart [file 41598_2018_27307_MOESM1_ESM.pdf]

A seven-lncRNA signature predicts overall survival in esophageal squamous cell carcinoma

Yu Mao[1]*, Zhanzhao Fu[1], Yunjie Zhang[1], Lixin Dong[1], Yanqiu Zhang[1], Qiang Zhang[1], Xin Li[1], Jia Liu[2]

1. Department of Oncology, First Hospital of Qinhuangdao, Qinhuangdao, Hebei.

2. Institute of basic medical sciences, Qilu Hospital, Shandong University, Shandong

*Corresponding author. Address: Department of Oncology, The First Hospital of Qinhuangdao, Wenhua Road No. 258, Haigang District, Qinhuangdao, Hebei, China. Tel.: +86 13333282619.

E-mail address:ayabluewin@163.com

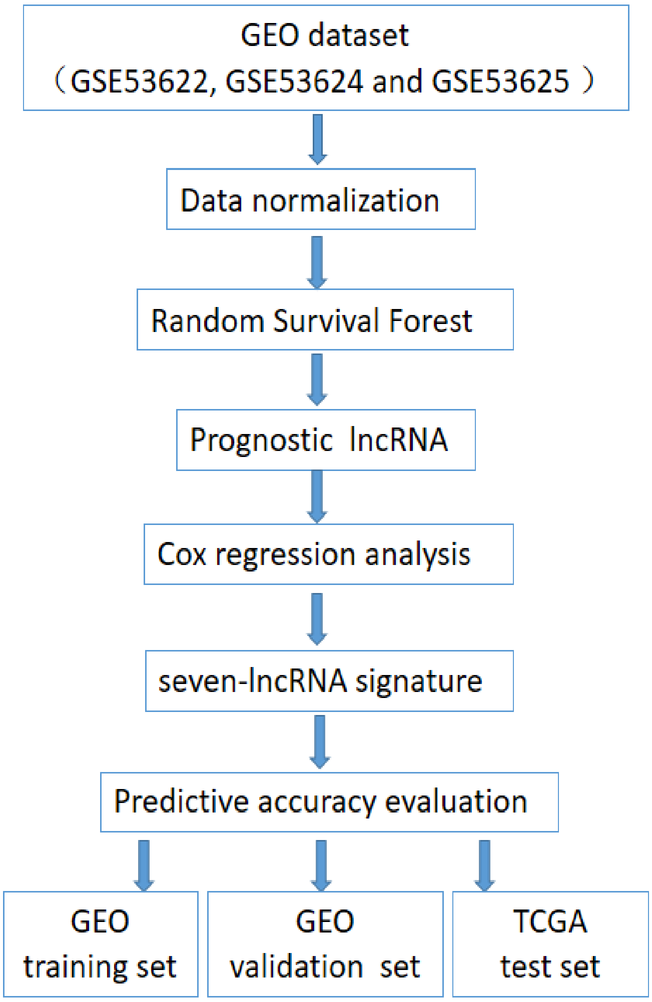
GEO dataset
（GSE53622, GSE53624 and GSE53625 ）
Data normalization
Random Survival Forest
Prognostic lncRNA
Cox regression analysis
seven-lncRNA signature
Predictive accuracy evaluation
GEO training set
GEO validation set
TCGA test set
